# Stereotactic body radiation therapy for abdominal oligometastases: a biological and clinical review

**DOI:** 10.1186/1748-717X-7-126

**Published:** 2012-08-01

**Authors:** Mohammed Yahia Almaghrabi, Stéphane Supiot, Francois Paris, Marc-André Mahé, Emmanuel Rio

**Affiliations:** 1 Department of Radiation Oncology, Integrated Oncology Centre, BD du Professeur Jacques MONOD, 44805, Saint-Herblain, France; 2 INSERM UMR, Nantes-Angers Oncology Research Centre, 8 Quai Moncousu, BP 70721, 44007, Nantes cédex 1, France

**Keywords:** Cancer, Gastrointestinal, Liver, Radiotherapy, Radiation biology, Surgery

## Abstract

Advances in imaging and biological targeting have led to the development of stereotactic body radiation therapy (SBRT) as an alternative treatment of extracranial oligometastases. New radiobiological concepts, such as ceramide-induced endothelial apoptosis after hypofractionated high-dose SBRT, and the identification of patients with oligometastatic disease by microRNA expression may yet lead to further developments. Key factors in SBRT are delivery of a high dose per fraction, proper patient positioning, target localisation, and management of breathing–related motion. Our review addresses the radiation doses and schedules used to treat liver, abdominal lymph node (LN) and adrenal gland oligometastases and treatment outcomes. Reported local control (LC) rates for liver and abdominal LN oligometastases are high (median 2-year actuarial LC: 61 -100% for liver oligometastases; 4-year actuarial LC: 68% in a study of abdominal LN oligometastases). Early toxicity is low-to-moderate; late adverse effects are rare. SBRT of adrenal gland oligometastases shows promising results in the case of isolated lesions. In conclusion, properly conducted SBRT procedures are a safe and effective treatment option for abdominal oligometastases.

## Introduction

Stereotactic Body Radiation Therapy (SBRT) was developed in the wake of Intracranial Stereotactic Radiosurgery (SRS) and Fractionated Stereotactic Radiotherapy (SRT) as a result of technological advances made in the early 1990’s in tumour motion quantification and image guidance [1]. Stereotaxy is a form of neurosurgery that uses a mechanical head frame and a precise 3-dimensional (3D) coordinate system to align and direct surgical instruments. SRT uses the methods of stereotactic neurosurgery to locate and target malignant and benign brain lesions before the delivery of radiation therapy [2].

In 1994, Lax et al. in Sweden applied this 3D approach to the targeting of extracranialtumours [3]. They constructed a combined body frame-abdominal compressing device and devised a method for the placement of external fiducial markers that could be indexed to the internal target. In 1998, Uematsu et al. developed a frameless “focal unit” which combines a linear accelerator (Linac), computed tomography (CT) scanner, and X-ray simulator (X-S) [4]. Treatment of 66 primary and metastatic lung carcinomas by this technique resulted in only 2 cases of local progression.

SBRT is a superior “focal unit” combining hypofractionated multi-beam conformal radiotherapy (CRT) and image-guided radiotherapy (IGRT). It delivers a high radiation dose, divided into several fractions (“hypofractionated”), to extracranial lesions. It is a high- precision technique with tight planning margins and a sophisticated treatment plan allowing rapid dose fall-off away from the treatment area. It provides improved volume targeting and smaller irradiated volumes of normal tissue. Its use is usually limited to well-circumscribed tumours (maximum cross-sectional diameter of up to 5 cm) but tumours as large as 7 cm have been treated. Low isodoses (e.g. 80% isodose) are often prescribed due to dose heterogeneity within the target [5]. Its exact definition can, however, vary.

SBRT use was initially confined to patients with unresectable or medically inoperable tumours. Nowadays, however, its use has been extended to patients with resectable or medically operable tumours. Results are encouraging in the treatment of lung and liver metastases but less clear-cut in the treatment of abdominal lymph node (LN) and adrenal gland. Provided that dosimetric constraints are met, SBRT is well tolerated.

Oligometastases are metastases that are limited in number and location. Hellman and Weichselbaum coined this term in 1995 [6]. They hypothesised that patients with less aggressive tumours and few new metastases during the first 4 months of first metastatic progression could potentially benefit from metastasis-directed therapy. They also identified a class of small RNAs, known as microRNAs, which might help distinguish patients with stable oligometastatic disease from patients with progression to polymetastatic disease. Oligometastases produced by early progression of primary lesions are known as *de novo* “oligometastases” whereas widespread metastases correspond to a state of “induced oligometastases” [7].

The aim of SBRT is to achieve good local control (LC) of each oligometastatic site and possibly the cure of some patients. Careful patient selection is needed. The best candidates for SBRT are patients with controlled primary tumours (colon or breast, sarcomas, kidney carcinomas), 4 or fewer oligometastatic sites, oligometastases < 5 cm, younger age, and good performance status [1].

This review will present the emerging role of hypofractionated SBRT in treating oligometastases to abdominal organs and lymph nodes.

### Biology of hypofractionated SBRT and oligometastases

Experimental work has suggested that the greater efficacy of hypofractionated high-dose SBRT over normofractionated RT in the treatment of radioresistanttumours might be due to different effects on both tumour and normal cells.

Radiation-induced cell death has been modelled according to the linear-quadratic (LQ) model which postulates that multiple radioinduced lesions interact in the cell to trigger cell killing. This assumption is described by the equation: S = e-(aD + bD2). The model is widely used in RT to calculate isoeffect doses for different.

According to the LQ model, high-dose fractions are needed to enhance tumour cell death. Treatment planning margins must therefore be small in order to reduce the total volume of normal tissue within the radiation field and to minimise late adverse effects, and the interval between fractions must enable complete repair of normal tissues. The LQ model is a reliable and mechanistically plausible model for designing protocols in the 2–10 Gy dose per fraction range. A loss in accuracy is expected above 10 Gy but, according to animal data, the model remains acceptable for designing clinical trials up to a 15–18 Gy dose per fraction [8].

The concepts underlying the damaging effects of ionising radiation on normal tissue architecture were introduced in the 1980’s [9,10]. Basically, organs are divided into two types according to their response to RT: (i) parallel functioning organs are made up of functional subunits that act independently (lung, liver, kidneys, and glandular tissues beyond the major ducts); (ii) serial functioning organs are made up of functional subunits that act in cooperation (bowels, bronchi, large ducts in glands, blood vessels, and nerves). SBRT can cause profound late adverse effects if delivered near serially functioning tissues [1].

The biologically most significant mechanism underlying radiation-induced damage in tumours and normal tissue is generally thought to be clonogenic cell death due to radioinduced DNA double-strand breaks (DSBs). Recently, however, ceramide-induced endothelial apoptosis rather than DNA DSB-induced clonogenic cell death has been considered to underlie the effects of hypofractionated SBRT [11]. Protection of endothelial cells against apoptosis induced by exposure to clinically relevant radiation doses is given by basic fibroblast growth factor (bFGF), and this natural protection mechanism may be associated with radiation resistance in normal and malignant tissues in vivo [12]. An immune-mediated mechanism might also contribute toward better survival outcomes for SBRT than conventional RT.

Experimental work has shown that ablative RT (15–25 Gy × 1) alone generates strong enough CD8+ T cell–dependent immunity to lead to tumour reduction, reduced relapse of primary tumour, and even eradication of metastasis in some settings [13], thus bringing current intensive RT/chemotherapy protocols into question. Finally, microRNAs may play a key role. A study of 42 tumour samples from oligometastatic patients treated with high-dose RT found that microRNA-200c expression enhancement in an oligometastatic cell-line resulted in polymetastatic progression [14]. The radiobiology of high-dose RT delivered to oligometastases is a field of intensive study.

### The SBRT procedure

SBRT has to overcome four hurdles: (i) patient positioning, (ii) internal organ motion, (iii) target volume shrinkage or expansion and (iv) subclinical malignant involvement not identifiable on the images available at treatment planning [15]. Proper patient positioning, target localisation, and management of breathing–related motion are essential to ensure the tight planning margins of SBRT. SBRT uses a wide variety of imaging techniques to delineate lesions and in dose calculations, of patient immobilisation and positioning devices to ensure safety and reliability, and of image guidance systems (Table 1) [5,16]. Recent SBRT-ready machines integrate several state-of-the-art RT capabilities (IGRT and immobilisation and respiratory motion solution technology) into a single machine.

**Table 1:** Components of SBRT

**Component**	**Some examples**
Imaging	General radiography; 3- or 4-Dimensional Computed Tomography (CT); Magnetic Resonance Imaging (MRI); Magnetic Resonance Spectroscopy (MRS); Positron Emission Tomography (PET) with or without image fusion with other techniques
Patient immobilisation and positioning	Stereotactic Body Frame™ (Elekta, USA); BodyFix™ (Medical Intelligence, Germany)
Image guidance	SonArray™modular ultrasound unit (Zmed, Ashland, USA); ExacTrac™ Ultrasound Localisation (BrainLAB, USA); BAT™ system (Nomos, USA).
SBRT-ready machines	Novalis™, BrainLAB, USA; Electa’s Synergy™; Varian’s Triology™; Tomotherapy’sHiArt (Tomotherapy, USA); Cyberknife™ (Accuray, USA), VERO-SBRT™ (Germany).

On completion of pretreatment simulation, the Gross Tumour Volume (GTV) is outlined in each slice where the lesion appears. A Clinical Target Volume (CTV) is applied to account for microscopic extension of the lesion although, in the case of metastases, the CTV and GTV are considered equal. The final Planning Target Volume (PTV) has even larger margins (≤10 mm) in order to correct for inaccuracies in the delivery system [17,18]. In general the PTV is encompassed by the 65-95% isodose line. SBRT may use a single treatment fraction (“radiosurgery”) or up to 10 fractions (“hypofractionated” SBRT).

Most reports on SBRT delivered by a gantry-operated linear accelerator mention the use of a method of motion management at planning and/or treatment. A variety of methods are used: (i) addition of constraints to the immobilisation device (e.g. dual vacuum technology for Medical Intelligence’s BodyFix™, a diaphragm control device for Elekta’s Stereotactic Body Frame™), (ii) use of tracking devices with fiducial markers implanted near the target volume (e.g. Cyberknife™, BrainLabExacTrac™), (iii) Gated Radiotherapy (GRT). Internal fiducial markers are used to track tumour motion in real-time during treatment but can cause artefacts on the images used for treatment planning. Although SBRT is considered to be non-invasive, these markers are implanted percutaneously under CT- or ultrasound-guidance. Their purpose is to reduce internal organ motion to < 5 mm.

The efficacy of GRT was first established on treatment of tumours located close to the diaphragm [19]. In GRT, devices that monitor breathing trigger radiation delivery during specific phases of the respiratory cycle, or the radiation beam is continuously turned on and off to synchronize delivery with the respiration cycle. Patients can breathe freely (e.g. Varian’s Real-time Position Management™ (RPM) system) or a pause in breathing can be induced either by an occlusion valve operated by the clinician (e.g. Elekta’s Active Breathing Coordinator™ (ABC)) or voluntarily by the patients themselves (deep inspiratory breath hold (SpiroDynr’X™, France). A breath-hold device is used with single-shot echo-planar-imaging (EPI) but also with other types of IGRT such as 3D-CBCT.

Geometrical uncertainties, which can be machine related (e.g. laser misalignment) or patient related (e.g. target volume definition, setup errors, organ motion), are handled by applying safety margins (Table 2) [20-25]. In addition, basing nodal RT portals on vascular rather than bony anatomy can significantly reduce normal tissue irradiation [26]. IGRT enables automatic correction of patient position through translation and rotation of the treatment couch on based measurements provided by the imaging system [27]. The most common image guidance system used to obtain volumetric information on patient geometry is 3D-cone-beam-CT (CBCT). It has become standard equipment on many modern linear accelerators.

**Table 2:** Advocated safety margins

**Team**	**Metastases**	**Recommendation**
Schefter et al. 2005 [20]	SBRT of liver metastases	Minimum distance between GTV and PTV surfaces:
		— 0.5 cm, axial planes
		— 1.0 cm, superior/inferior
		Cumulative maximum tumourdiameter: < 6 cm
Katria et al. 2010 [21]	IGRT of abdomen and pelvis	Stroom's and Van Herk's margins (cm):
		— 0.39 and 0.35 mm, anterior-posterior
		— 0.94 and 0.46 mm, medial-lateral
		— 0.40 and 1.09 mm, superior-inferior
Wysocka et al. 2010 [22]		Median 3.8 mm intrafraction craniocaudal displacement for coeliac axis with smaller displacements for other axes
RTOG consensus guidelines [23]	Inguinal lymph nodes	Caudad extent of the inguinal region : 2 cm to the saphenous/femoral junction
Kim et al. 2011 [24]	Inguinal and femoral nodes	2.2 - 2.9 cm around femoral vessels
Van Weieringen et al. 2011 [25]	Pelvic lymph nodes	For offline and online correction protocols, respectively:
		— 7 and 5 mm, left-right
		— 6 and 5 mm,craniocaudal
		— 8 and 7 mm,dorsoventral

RTOG: Radiation Therapy Oncology Group.

Biological image-guidance and dose-painting are two developments that have been combined with SBRT to customise treatment. However, they have not yet been validated for SBRT of oligometastases. Ling et al. hypothesised that “biological” images revealing metabolic, functional, and physiological activity can be used to derive a biological target volume (BTV) in order to incrementally improve target delineation and dose delivery [28]. For example, image fusion between MRI and FDG-PET/CT in a simulator is used to identify hypermetabolic activity areas and microscopic disease extension. MRI tends to be used for imaging of liver oligometastases and PET for imaging of abdominal LN and adrenal oligometastases. Fluoro-D-glucose (FDG)-PET/CT use is hampered, in the case of liver oligometastases, by breathing-related motion and physiological FDG uptake by the liver and, in the case of abdominal oligometastases, by peristalsis movements of the intestine. Dose-painting radiotherapy (DPRT), which makes use of PET-CT with tracers other than FDG (fluoromisomidazole (Fmiso) or fluroro-L-thymidine (FLT)) or dynamic contrast enhanced (DCE)-MRI, delivers a higher dose to the most radioresistant areas of the tumour and reduces the dose to the most sensitive areas [29].

Although practice guidelines on SBRT have been issued by the American Society for Therapeutic Radiology and Oncology (ASTRO) and the American College of Radiology (ACR), wide variations persist among centers in prescription patterns and treatment delivery systems [16].

### Methodology of systematic review of clinical trials on SBRT of oligometastases

Most published work on SBRT use in oligometastatic abdominal disease concerns liver metastases rather than abdominal lymph nodes or adrenal gland metastases.

The following summary of clinical trials is based on a systematic Medline search (languages: English & French; key-words: “stereotactic”, “radiotherapy”, “cancer: metastases, oligometastases”, “liver”, “abdominal lymph nodes”, “adrenal gland”, “extracranial”, “surgery”; publication types: original articles and reviews; dates: 1995 to Nov 2010; updated in Mar. 2012 and of a manual search of cited references, selected journals, and abstracts of international congresses (ESTRO2010, ASTRO2010 and ASTRO2011). Preferential article selection criteria were as follows: (i) for SBRT of liver metastases: prospective trials reporting actuarial local control (LC) in ≥20 lesions after treatment with ≤5 SBRT fractions, as well as trials on primary liver cancer and oligometastases, (ii) for SBRT of abdominal LN and adrenal gland metastases: all available prospective and retrospective studies trials. Because of marked variations in the reporting of doses and outcomes among studies, a unified analysis was not possible.

### SBRT of liver oligometastases

The liver is a common metastatic site for a variety of primary malignancies including colorectal, lung, breast, bladder, oesophageal, head & neck and pancreatic cancers, and cholangiocarcinoma. According to the most recent version of the National Comprehensive Cancer Network **(**NCCN) guidelines (v.3.2012), surgical resection is still the standard of care for liver metastases. However, despite the favourable outcomes that have been reported after surgery [30,31], 80-90% of patients are either patients with lesions that are surgically not resectable or are medically inoperable patients at the time of diagnosis [32].

The first radiotherapeutic modality used to treat liver metastases was Whole Liver RT (WLRT) [33]. Results were encouraging but radiotherapy regimens of 32 Gy in 16 fractions or more were insufficient to eradicate disease and were often associated with radiation-induced liver disease (RILD). RILD is a clinical syndrome of anicteric hepatomegaly, ascites, and elevated liver enzymes occurring within 3 months after completion of therapy. Attempts to increase the efficacy of WLRT proved unsatisfactory [34] and led to the development of Partial Liver Radiation Therapy (PLRT). Low-dose WLRT nevertheless remains a useful treatment for symptom palliation in patients with end-stage cancer and diffuse metastatic infiltration of the liver which has become refractory to systemic treatment [35]. Outcomes for PLRT, which is usually a non-targeted therapy, were better than for WLRT but not lasting. The 6-month actuarial LC of 62% for PLRT is, however, far below the most recent LC rates recorded for SBRT [36].

Compared to WLRT and PLRT, SBRT has the advantage of delivering higher tumoricidal doses to the target and sparing uninvolved liver and surrounding critical organs, thus diminishing the likelihood of RILD. SBRT provides more accurate radiation delivery, meets the normal tissue constraints better (Table 3) [37,38], and has a smaller PTV margin. The initial LC rate for SBRT of liver metastases was 80% back in 1995 [39]. However, the latest reports withbetter patient selection show higher efficacy and lower toxicity as well as improved outcomes from advances in multimodality-imaging.

**Table 3:** Recommended dose constraints to the liver

**Whole-liver RT (WLRT)**	**Partial liver RT (PLRT)**	**Stereotactic body radiation RT (SBRT)**
≤ 30 Gy, 2 Gy/fraction	< 32 Gy, 2-Gy/fraction	< 15 Gy in 3 fractions
21 Gy in 7 fractions	At least 10% of normal liver spared from radiation	< 20 Gy in 6 fractions
		≥ 700 mL of normal liver receives ≤ 15 Gy
		3 to 5 fractions

### Patient selection

Strict selection of patients is required to limit normal tissue toxicity to intra-abdominal organs including the liver, stomach, and duodenum. For inclusion in our systematic review, patients had to present no more than 3 metastases; no lesion >6 cm; no lesion immediately adjacent to the GI tract (distance >6 mm) in radiosurgery patients; and adequate pre-treatment baseline liver function. The RT regimen had to be ≤ 5 fractions of radiation, with ≥700 mL of normal liver receiving ≤15 Gy (for 3 fractions) or at least 700 mL for a cumulative dose of 21 Gy (for 5 fractions). GTV was expanded by 5–10 mm to yield the PTV. These criteria for radiation dose, however, are not derive from validated guidelines but are suggestions based on radiobiological modelling or expert opinion.

On applying these selection criteria, we retrieved 10 prospective studies with a wide variety of doses and schedules (Table 4) [40-49]. The highest total dose per fraction was 60 Gy. The number of fractions was usually 3 (range, 1 to 5). Centrally located lesions might require delivery of the highest number of fractions because of the proximity of critical structures [49].

**Table 4:** SBRT of liver oligometastases

	**Liver metastases (N)**	**Dose (Gy × fr)**	**Median follow up (mos)**	**Actuarial LC rate (%)**	
				**at 12 mos**	**at 24 mos**
Herfarth et al. 2001 [40]	56	14-26 **×** 1	6	75	
Wulf et al. 2001 [41]	23	30 **×** 3	9	76	61
Wulf et al. 2006 [42].	51	variable dose **×** (1–3)	15	92	66
Hoyer et al. 2006 [43]	97/141*lesions	45 **×** 3	4.3 years		79
Méndez-Romero et al. 2006 [44]	34	37.5 **×** 3	13	100	86
Kavanagh et al. 2006 [45].	36	60 **×** 3	19	93 (at 18 mos)	
Milano et al. 2008 [46].	120/293*lesions	50 **×** 5	41		67
Rusthoven et al. 2009 [47].	63	36 - 60 **×** 3	16	95	92
Van der Pool et al. 2010 [48]	31	37.5 - 45 **×** 3	26	100	74
Rule et al. 2010 [49].	37	30 **×** 3	20		56
		50 **×** 5			89
		60 **×** 5			100

LC: local control.

* Ratio of liver oligometastases to total number of oligometastases.

### Local control and follow-up

Dose escalation, tested by 4 institutions, was associated with improved LC and median survival rates. For liver SBRT as for lung SBRT, a higher radiation dose and a smaller GTV were significant predictors of better LC in univariate analyses. The 3-year actuarial LC rate was 89.3% for a nominal dose of ≥54 Gy compared to 59.0% for a 36–53.9 Gy dose and 8.1% for a <36 Gy dose [50]. Among the 10 selected trials in Table 4, doses ≥54 Gy were administered in 2 trials. Kavanagh et al. recorded LC rates of 100% and 93% at 1 and 2 years in 36 patients with liver metastases receiving 60 Gy in 3 fractions. Rule et al. observed excellent LC in their 60-Gy cohort, with no failures at 1 or 2 years. Poorer LC was recorded in trials using doses < 36 Gy (3 fractions) or 20 Gy (radiosurgery) [40,42]. Poorer LC was associated with a greater GTV. The 2-year LC rate was 100% for ≤3 cm lesions compared to 77% for >3 cm lesions (P = 0.015, log-rank test) [47]. In a study of 293 lesions, multivariate analysis revealed a significant association between colorectal metastases and multiple local failure [46], but prior chemotherapy might account for this unconfirmed observation [49]. Overall, the foregoing findings suggest that patients with oligometastases ≤ 3 cm and who receive doses of 60 Gy might be most likely to achieve long-term control.

Follow-up of patients after liver SBRT is a challenge as the early treatment response (before 3 months) may be difficult to interpret on CT or MRI images because of a reaction to radiation. This reaction may be a form of veno-occlusive disease but is not associated with changes in overall liver function. Changes in contrast enhanced CT-scans were observed in all 44 patients of the Wulf et al. study after fractionated treatment with a cumulative 20 Gy-isodose [42].

LC after SBRT of liver oligometastases is sustained. A median time to maximal response of 6 months and a 71% LC rate (95% confidence intervals, 58–85) were reported in a Phase I study of 68 patients receiving 41.8 Gy (range, 27.7 to 60 Gy) [42]. A PET scan obtained at 13 months post-SBRT in an interim analysis of a Phase II study revealed marked uptake in the treated lesion [45].

### Toxicity

Most investigators have evaluated the toxicity of SBRT for liver oligometastases using the Common Terminology Criteria for Adverse Events (CTCAE v3.0). Three cases of fatal toxicity were recorded by the pioneers of liver SBRT in patients with large hepatocellular carcinomas and pre-existing liver disease (ascites, jaundice, cirrhosis, or hepatitis C) [3]. No SBRT-related deaths were reported in the studies we reviewed. In a study of 141 lesions with metastatic colorectal cancer including 69% with liver oligometastases, there was a death from liver failure in a patient receiving >10 Gy to 60% of the liver (median: 14.4 Gy) and a colon perforation warranting surgery [43].

Serious late SBRT-related complications are not uncommon. Late signs of liver fibrosis, portal hypertension, ascites and bleeding from oesophageal vertices have been described 28 and 41 months after irradiation of two targets close to the liver hilum [42]. A patient with a late episode of ascites (Grade 2) developed a portal hypertension syndrome with melena (Grade 3) [44]. Other possible late sequels have been a case of thoracic pain after irradiation of targets very close to the thoracic wall and a case of rib fracture 10 months after irradiation of a subcapsular liver metastasis located in the vicinity of the ribs [42,48]. Early complications reported by Mendéz et al. within 3 months of liver SBRT were elevated gamma-glutamyltransferase (Grade 3) in 3 patients, asthenia (Grade 3) and liver toxicity (Grade 2) in a previously treated patient (chemotherapy and resection) [44]. Ulcers and perforations were noted after delivery of >30 Gy in 3 fractions to the intestine [43]. Grade 3 gastritis and skin toxicity (oedema and breakdown) have also been reported [45]. Less serious adverse effects ranged from 29% in one study (Grade 1 or 2 fever, chills or pain, nausea and vomiting) to 38% in another (intermittent appetite or mild nausea, moderate singulatus, and fever) [40,42].

### SBRT of abdominal lymph node metastases (Local control and toxicity)

The rationale of administering abdominal SBRT with curative intent to patients with limited nodal metastatic disease is the same as for selected patients with liver or lung metastases. In the case of a single abdominal node, it has been suggested that elective SBRT should incorporate abdominal nodal chains and boosting pathological nodes. This is because a common pattern of relapse of abdominal lymph node (LN) metastases would seem to be within adjacent LNs [51]. Incorporating adjacent retroperitoneal (RP) LNs in the absence of concurrent distant progression was based on the observation that, whereas 6 of 11 patients with 22 oligometastases (19 abdomino-pelvic LNs) treated by hypofractionated IGRT progressed in new para-aortic/RP LNs outside the PTV, first failure in the other 5 patients included adjacent RP LNs at/near midline within 1–2 vertebral bodies of the treated LNs [52]. In this study, Grade 3 GI bleeding occurred in a patient with 3 periduodenal LNs receiving 3×8 Gy.

Six studies have investigated SBRT of abdominal LN oligometastases in a total of 118 inoperable patients with a variety of primary lesions (Table 5) [51,53-57]. There is no consensus on optimal dose, number of fractions, or planning constraints. The highest dose was 60 Gy; the number of fractions ranged from 3 to 6. Median follow-up was 12–28 months. PET-CT was used in 3 studies. Organ motion was minimised with a vacuum pillow or abdominal compression. In the largest study (41 patients), Bae et al. reported PFS, LC and OS rates for 18 colorectal cancer patients with LN metastases treated by SBRT (45–51 Gy in 3 fractions) (Cyberknife™) and followed up for a median of 28 months (range, 6–65) [51]. Three-year rates were 40%, 64% and 60% respectively, and 5-year rates were 40, 57% and 38%, respectively.

**Table 5:** SBRT of abdominal lymph node oligometastases

	**Patients (N)**	**Primary cancer**	**Dose (Gy) × fr**	**Median follow up (mos)**	**Outcomes**
Jereczek-Fossa et al. 2009 [53]	14	Prostate	33 (mean) **×** 3-5	Mean 18.6	- No in-field clinical progression
					- Distant or regional LN progression at mean time of 12.7 mo
					- All patients with relapse had high-risk disease
Bignardi et al. 2011 [54]	19	CRC (5/19)	45 **×** 6	12	Actuarial rate of freedom from local progression: 77.8 ± 13.9 at both 12 and 24 mos
					Minimal acute and chronic toxicity
Choi et al. 2010 [55]	30	Uterus and cervix	EBRT: 27–45 (n = 4 pts)	15	4-year LC rate: 67.4%
			SBRT: 33–45 **×** 3 (n = 24 pts)		4-year OS rate: 50.1%
					(all 30 pts).
Kim et al. 2009 [56]	7	Gastric (salvage aftersurgery)	48 (median) **×** 3	26	Complete response: n = 5
					Partial response: n = 2
Kim et al. 2009 [57]	7	CRC	Escalated dose 36–51 **×** 3	26	Median survival: 37 mos
					1-year OS: 100%
					3-year OS: 71.4%
					G4: intestinal obstruction in 1/7 patients
Bae et al. 2012 [51]	41	CRC	48(45 – 60) **×** 3	28	-PFS, LC and OS rates
					3-year rates : 40%, 64%, 60%
					5-year rates : 40, 57%, 38%
					-G3 perforation after pelvic LN SBRT;G4 obstruction of para-aortic LN SBRT

EBRT: electron beam radiotherapy; CRC: colorectal cancer; LN: lymph node; LC: local control, OS: overall survival, G: grade.

A GTV of ≤17 ml and early radiological response and were favourable predictive factors for LC. The 4-year LC rate was significantly higher for complete responders vs poor responders or non-responders (90% vs 24%, p = 0.014) and in patients with a GTV of ≤17 mL (P = 0.0059) [51,55]. In a univariate analysis, the number of metastases was the only significant prognostic factor identified for 2-year PFS) (41.7% (solitary) vs 0% (nonsolitary), p <0.0004) [54].

In all 6 studies, three patients experienced serious adverse effects (Grade 3 perforation after pelvic LN SBRT (51 Gy) needing colostomy; 2 patients had Grade 4 intestinal obstruction of para-aortic LN SBRT (48 Gy) needing small bowel resection). In the second largest study (30 patients), 5 of the 25 patients receiving concomitant chemotherapy developed Grade 3 or higher acute haematologic toxicity during chemotherapy [55]. One of the 30 patients developed a urethral stricture 20 months post-SBRT.

In brief, outcomes after SBRT for abdominal LN metastases were similar to those obtained after surgery. Optimal normal tissue constraints and acceptable toxicity have not yet been established.

### SBRT of adrenal gland oligometastases

Adrenal gland metastases from non–small cell lung carcinoma (NSCLC) are present in 5 to 10% of patients at initial presentation. Surgical resection is the main treatment option. In a review of 11 studies in a total of 60 lung cancer patients who underwent surgical resection of adrenal metastases, median survival was 14–24 months [58]. In patients with an isolated adrenal metastasis, the 5-year OS rate ranged from 10 to 23% [59]. However, according to Porte et al., aggressive operative intervention is not indicated in patients with a solitary synchronous contralateral metastasis and operable NSCLC as these metastases are likely to be the first manifestation of disseminateddisease because they develop mainly by the haematogenous route**.** They prefer restaging 3 months after lung resection instead. If the metastasis is homolateral to the primary lesion, a synchronous complete removal can be performed without any added morbidity or mortality [60]. For metachronous metastases, a minimum 10-month interval between diagnosis and resection of metastases is advocated to ensure that there are no other metastatic sites. Patients with a shorter interval between lung resection and adrenal metastasis diagnosis may be amenable to RT or chemotherapy [61].

Clinical experience relating to SBRT of adrenal metastases is limited. We retrieved 4 retrospective studies in a total of 130 patients with a variety of primary lesions, mostly NSCLC (Table 6) [62-65]. The highest dose was 50 Gy; the number of fractions ranged from 3 to 10. Median follow-up was 9.8-41 months. The low LC rate, reported by Chawla et al. was due to the inclusion of 16/30 patients who underwent SBRT for palliation or prophylactic palliation of bulky adrenal metastases [62]. On the other hand, the median OS of 23 months reported by Holy et al. for patients with an isolated adrenal metastasis is similar to that for surgical resection [63]. According to Casamassima et al., SBRT may be considered as an ablative therapy that is not influenced by factors such as the primary tumour, type of oligometastatic adrenal disease (synchronous vs. metachronous, unilateral vs bilateral) and PTV. Treatments were generally well tolerated. Definitive end-points for toxicity have not been established. A case of Grade 2 adrenal insufficiency was reported by Casamassara et al. [64].

**Table 6:** SBRT of adrenal gland metastases

	**Patients (N)**	**Median dose (Gy)**	**Median follow up (mos)**	**Outcomes**
Chawla et al., 2009 [62]	30	40 Gy (16–50)/4–10 fractions	9.8	At 1-year: survival: 44%, LC: 55%, distant control rate, 13%
				No late Grade ≥2 toxicity
Holy et al., 2011[63]	18	20-40 Gy/5 fractions	21	In 13 patients with isolated adrenal metastasis: LC :77%, OS:23 months
Casamassima et al., 2012 [64]	48	36 Gy/3 fractions^a^	16.2	At 1 and 2 years, LC: 90%; OS: 39.7% and 14.5%, resp.
				1 case of Grade 2 adrenal insufficiency
Scorsetti et al., 2012 [65]	34	32 Gy/4 fractions	41	At 1 and 2 years: LC 66% and 32%, resp.
				No significant acute and late toxicities

^a^70% isodose, 17.14 Gy per fraction at the isocenter. Eight patients were treated with single-fraction (23 Gy) stereotactic radiosurgery.

A retrospective comparison by matched case–control study, of surgery (laparoscopic adrenalectomy) versus SBRT (36 Gy in 5 fractions) of an isolated adrenal oligometastases in patients with controlled primary tumours revealed no significant difference in survival at 6 months and 1 year (77% (95% CI: 49–100) and 62% (26–97), respectively, for SBRT and 87% (75–98) and 77% (64–91) for LA) [66]. However, these findings need to be confirmed in a prospective study with longer term follow up.

In conclusion, SBRT may be an alternative to surgical resection, especially for solitary adrenal metastases, and displays a low toxicity profile. However, because toxicity occurs late, close attention needs to be paid to the length and quality of follow-up.

### Looking ahead

Until fairly recently, RT of metastases was considered to be palliative only. The above recent advances in imaging and biological targeting, however, provide support for SBRT with curative intent in an oligometastatic setting although, as yet, there is no consensus on schedule (optimal doses, number of fractions, and treatment delivery accuracy). The efficacy of SBRT is well established for liver oligometastases but less well established, although promising, for abdominal LN metastases (one 5-year study) and adrenal gland oligometastases (survival similar to surgical resection for solitary adrenal metastases). Further prospective studies are needed to confirm these results.

SBRT is more effective in the case of “*de novo* oligometastases” than widespread metastases. A state of “induced oligometastases” needs effective systemic therapy to eradicate most metastatic sites. Emphasis should therefore be placed on the criteria needed for effective patient selection and on delivery of appropriate therapy. Approaches such as microRNA detection have shown promise in patient selection.

Distant metastases are the main cause of death after SBRT. Chemoradiotherapy thus deserves study. Chemotherapy increases organ sensitivity to radiation and also kills tumour cells disseminated in the blood. Promising antitumour responses without potentiation of RT toxicity were observed in a Phase I study of concurrent sunitinib and hypofractionated IGRT followed by maintenance sunitinib in patients with oligometastases [67]. A multi-institutional Phase II trial is ongoing. In 2007, we initiated a Phase II prospective multicentre study of SBRT and concurrent irinotecan in colorectal cancer patients with unresectable liver and lung metastases. As preliminary results for the combination did not differ significantly from those for SBRT alone, we increased the tumoricidal dose in order to achieve higher LC [68]. The study is ongoing.

In conclusion, published studies suggest that SBRT is a valuable alternative to surgery in patients with liver or abdominal lymph node oligometastases although no SBRT standards have yet been defined for the latter. The value of SBRT in the treatment of adrenal oligometastases will depend on their early detection. Future attempts to improve outcomes could focus on including a SBRT plus chemotherapy arm in SBRT trials and identifying patients with oligometastatic disease by microRNA expression.

## Competing interests

The authors declare that they have no competing interests.

## Authors’ contributions

RE is responsible for the version submitted for publication. MMA planned the structure of the article and critically read the manuscript. SS and PF critically read the manuscript and wrote the section on the biology of hypofractionated SBRT and oligometastases. AMY performed the critical review of the literature and drafted the manuscript. All authors read and approved the final manuscript.
